# Protocol for 4D live fluorescence microscopy to image rapid cellular responses in yeast

**DOI:** 10.1016/j.xpro.2025.104026

**Published:** 2025-08-13

**Authors:** Yaneris M. Alvarado-Cartagena, Christopher Huppenbauer, Nava Segev

**Affiliations:** 1 Department of Biochemistry and Molecular Genetics, College of Medicine, University of Illinois Chicago, Chicago, IL, USA; 2 Advanced Imaging and Research Applications W. Nuhsbaum, Inc., McHenry, IL, USA

**Keywords:** Cell Biology, Microscopy, Molecular Biology

## Abstract

Micro-autophagy is thought to occur through cargo engulfment by the lysosomal membrane based on electron microscopy of fixed cells. We present a protocol that follows micro-ER (endoplasmic reticulum)-phagy in live yeast cells that can be used for following other rapid cellular processes. We describe steps for inducing the accumulation of misfolded proteins and their selective shuttling for degradation during nutritional stress. We then detail procedures for live-cell time-lapse confocal microscopy and 3D reconstruction showing individual events of cargo engulfment and internalization into the lysosome that occur within seconds.

For complete details on the use and execution of this protocol, please refer to Gyurkovska et al.^1^

## Highlights

• Steps for live-cell confocal microscopy imaging of micro-ER-phagy in yeast
• Instructions for growing and stressing cells with fluorescent cargo and lysosomes
• Guidance on 4D visualization of cargo engulfment and internalization by the lysosome
• Procedures for achieving high temporal and spatial resolutions of rapid cell events

## Abstract (editor highlights)

Publisher’s note: Undertaking any experimental protocol requires adherence to local institutional guidelines for laboratory safety and ethics.

## Graphical abstract

**Figure:**
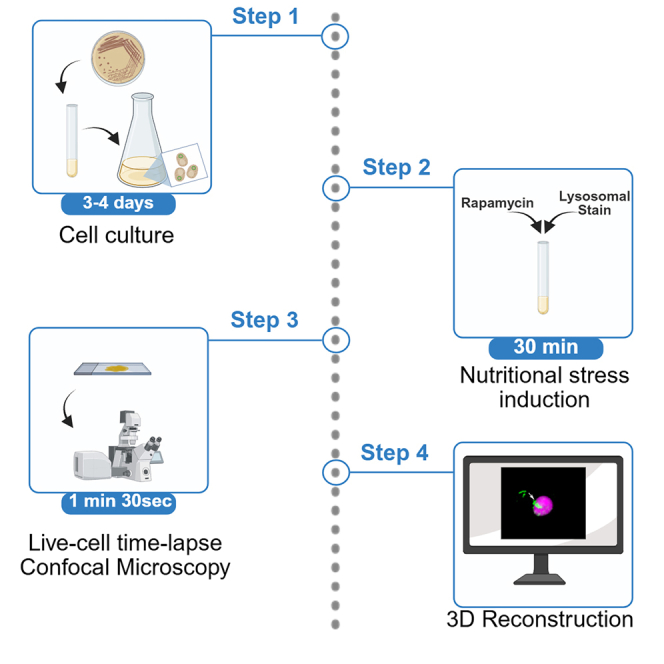

## Before you begin

The following protocol describes the steps of cell preparation, video capture, and analysis of micro-autophagic events in live budding yeast *S. cerevisiae* using 3D time-lapse, using a spinning disk fluorescence confocal microscope.^1^

### Micro-ER (endoplasmic reticulum)-phagy

Previous reports documenting micro-autophagy used electron microscopy of fixed cells, which can only suggest the steps of the process without giving information about dynamics and speed.^2^ To overcome these limitations and observe dynamic events, we used live cells, two fluorophores (for the cargo and lysosome), and 3D time-lapse microscopy. Due to the size of the lysosome and the unknown speed of the event, we needed to consider spatial and temporal resolutions.

### 3D time-lapse microscopy acquisition

To obtain good *spatial resolution*, high-speed fluorescence spinning disk confocal microscopy allows acquisition of Z-stacks of optical slices, which in turn allow 3D reconstruction of the structure. *Temporal resolution* depends on the speed of the Z-stack acquisition, which in turn depends on the time it takes to change the lasers and filters, especially if more than one fluorophore is used. Therefore, to image micro-autophagic events, we used two setups: for two fluorophores (cargo and lysosome), we used dual-channel imaging (using two discrete laser excitation wavelengths), whereas for a single fluorophore (lysosomal membrane), single-channel imaging (using a single laser excitation wavelength) was used. The latter is faster because there is no need to change lasers and filters.

### Using two alternative fluorophores for the lysosome

It is always preferred to use two alternative approaches for establishing novel concepts. Therefore, we used two fluorescent dyes to label the lysosome: FM-464 for the lysosomal membrane, and CMAC for the lysosomal lumen. Importantly, these two dyes also allowed us to use different kinds of dual-channel setups when using them with the fluorescent cargo: with a single dual-band (faster) or two emission filters (slower).

For our project, we describe the choice of cargo, cells, and fluorophores:

*The cargo*: Deg1-Vma12-GFP (expressed from MET25 promoter) is a fusion protein containing the Deg1-degron, which is cleared in wild-type cells by the ER-associated protein degradation (ERAD-C) through the E3 ligase Doa10, the integral membrane protein Vma12, and GFP. Deg1-Vma12-GFP accumulates in *doa10Δ* cells.^3^

***Note:*** The plasmid was constructed by Ravid *et al.*, the MET25 promoter was used in a previous study.^3^ For our purpose, the basal level of the promoter was used under growth in YPD.

*The choice of Saccharomyces cerevisiae cells with MHY8293 background*^1^^,^^3^*: doa10Δatg1Δ* expressing the cargo. The deletion of *DOA10* ensures that the cargo accumulates in the cells during normal growth. The *ATG1* deletion prevents macro-autophagy, ensuring we follow micro-autophagy under stress. These deletions in the wild-type strain expressing Deg1-GFP were constructed using homologous recombination of PCR-amplified cassettes by replacing the genes with *kanMX* and *hygro* cassettes, respectively.

*The choice of fluorophores*: The cargo, Deg1-GFP, was tagged on the chromosome. For visualizing the lysosomal membrane, we used FM4-64 in Methods 1 and 3. In Method 2, CMAC was utilized for lysosomal lumen staining. Method 1 allows for imaging with two lasers, enabling simultaneous capture of both the lysosomal membrane and the cargo. Method 2, by using CMAC, increases acquisition speed by eliminating the need to change filters between lasers. Finally, Method 3 further enhances acquisition speed by using only a single laser to image the lysosomal membrane.

### Yeast media preparation

**Timing: 1–2 h**

1. Yeast Extract Peptone Dextrose (YPD) liquid media:
  a. Mix the following into a beaker:

**Table:** 

Reagent	Amount
Yeast extract	10 g
Bactor Peptone	20 g
Distilled water	Up to 950 ml
  b. Transfer the mixture from the beaker into a 500 mL bottle.
  c. Autoclave the bottle.
  d. After the autoclave, keep the bottle at room temperature.
  e. Before using the media, add 50 mL of 40% glucose into the bottle.
    ***Note:*** Liquid media can be prepared 1–2 days before starting the experiment and store at room temperature for up to a month.
2. YPD plates:
  a. Mix the following into a 2000 ml flask:

**Table:** 

Reagent	Amount
Yeast extract	10 g
Bactor Peptone	20 g
Bactor Agar	20 g
Distilled water	Up to 950 ml
  b. Autoclave the mixture.
  c. After the autoclave, add 50 mL of 40% glucose and mix with a magnetic stir bar until the mixture cools off before pouring into Petri dishes.
    ***Note:*** Pour the mixture into Petri dishes in a sterile area with a flame to prevent contamination. Avoid any air bubbles on the surface.
  d. Leave the plates to dry at RT for 1–2 days before storing them at 4°C.
    ***Note:*** The 40% glucose solutions should be sterile prior to use.

### Lysosomal dye stocks

3. FM4-64 stock solution preparation^4^:
  a. Dissolve 1 mg of FM4-64 in 1 ml of DMSO for a stock solution of 1.6 mM.
  b. Store at −20°C.

***Note:*** FM4-64 is light sensitive; avoid direct light and repeated freeze-thaw cycles.

4. CMAC stock solution preparation^5^:
  a. Dissolve 500 μg of CMAC in 237 μl of DMSO for a stock solution of 10 mM.
  b. Store at −20°C.

***Note:*** These lysosomal dye stocks can be prepared 1 day before the experiment.

## Key resources table

**Table:** 

REAGENT or RESOURCE	SOURCE	IDENTIFIER
**Chemicals, peptides, and recombinant proteins**		

Rapamycin	LC Laboratories	R-5000
FM4-64	Invitrogen	T3166
CMAC	Life Technologies	Y7531
Bacto Peptone	Becton Dickinson (BD)	211677
Difco Yeast Extract	BD	210929
Difco Agar	BD	214010

**Experimental models: Organisms/strains**		

*Matα leu2-3*,*112 ura3-52 his3Δ200 lys2-801trp1-1* *TRP1*::*Deg1-F-Vam12-yEGFP*	Ravid et al.^3^	N/A
NSY1962 *doa10Δ*::*KAN*	Lipatova et al.^6^	N/A
NSY2017 *atg1Δ::HYGRO doa10Δ*::*KAN*	Gyurkovska et al.^1^	N/A

**Software and algorithms**		

LAS X version 3.7.4	Leica Microsystems	11640687
GraphPad Prism	GraphPad Software, Inc.	https://www.graphpad.com/
3D BLIND Deconvolution Algorithm	AutoQuant by Media Cybenetics	11650865
LAS X 3D Render Engine v.3.7.4	Leica Microsystems	11640853

**Other**		

Microscope: Leica DMis	Leica Microsystems	11889113,11889073, 11889086,11889037, 11889032,11889023, 11889022,11522100, 11525207, 11525228
Objective lens: 63x Oil, 100x Oil	Leica	11506350, 11506325
Light source: Oxxius L4C Laser Launch 405/488/561/640 nm	Oxxius	https://www.oxxius.com/products/l4cc-combiner/
Camera: Prime 95B	Teledyne Photometrics	https://www.teledynevisionsolutions.com/products/prime-95b/?vertical=tvs-photometrics&segment=tvs
Confocal system: csu-x1 Spinning Disc confocal	Yokogawa	https://www.yokogawa.com/us/solutions/products-and-services/life-science/spinning-disk-confocal/csu-x1-confocal-scanner-unit/#Details
Filter: Chroma spinning disc beam splitter (dichroic) (mounted inside confocal)	Chroma	https://www.chroma.com/products/parts/zt405-488-561-640rpc?objectID=parts--7556&queryID=e58e467d8c6c72f9a4d5599b263dca38
Filter: Chroma 25 mm Emission filters, single filters ET460/50 m, ET525/50 m, ET605/52 m, ET700/75 m	Chroma	https://www.chroma.com/products/parts/zet405-488-561-647m
Filter: Chroma Multi-pass Emmitter ZET 405/488/561/647 m 25 m	Chroma	416773
Superfrost microscope slides white tab 3″ × 1″ x 1.0 mm	Fisher Scientific	12550123
Cover glass #1 size 18 × 18 mm	Electron Microscopy Sciences	63756-01
Petri dish size 100 × 15 mm	VWR	25384-342

## Materials and equipment

All materials used in this protocol are described in before you begin section of this protocol. See the key resources table for information on reagents and equipment used for cell preparation and imaging.

**Table:** Yeast extract peptone dextrose (YPD) liquid media

Reagent	Final concentration	Amount
Yeast extract	10 g/L	10 g
Bactor Peptone	20 g/L	20 g
Distilled water	N/A	Up to 950 ml
40% Glucose	2%	50 ml
**Total**	**N/A**	**50 mL**

Store at room temperature for up to a month.

**Table:** Yeast extract peptone dextrose (YPD) plates

Reagent	Final concentration	Amount
Yeast extract	10 g/L	10 g
Bactor Peptone	20 g/L	20 g
Bactor Agar	20 g/L	20 g
Distilled water	N/A	Up to 950 ml
40% Glucose	2%	50 ml
**Total**	**N/A**	**50 mL**

Store at room temperature or 4°C for up to 3 months.

### Microscope settings

• Microscope: Laser-Based Spinning Disk Confocal attached to Leica DMi8 Inverted Microscope.
• Camera: The current setup is a single camera system that acquires sequential channel imaging. Back-thinned Scientific CMOS (sCMOS) camera (95% Quantum Efficiency) for low-level fluorescence and to avoid photobleaching.
  ***Note:*** The Leica DMi8 Inverted Microscope has an encoded Z motor with 25 nm repeatability, thus negating the need for a piezo Z stage.
• Laser:
  ○ Green Channel (cargo) exposure/laser power/wavelength: 200 ms/50%/488 nm.
  ○ Near Red Channel (FM464) exposure/laser power/wavelength: 100 ms/20%/561 nm.
  ○ UV Channel (CMAC) exposure/laser power/wavelength: 100 ms/15%/405 nm.
• Filters:
  ○ Chroma 25 mm Laser Blocked Emission filters, single narrow band filters.
  ○ Chroma 25 mm Dual-band Laser Emission filter (ZET).
    ***Note:****Filter properties*: Our spinning disk system uses single mode coherent polarized lasers (Oxxius) that enter the CSU-X confocal head via an FC/AFC laser fiber. There are no excitation filters on the system. The dichroic (beam splitter) is as quad (405/488/561/640) band Chroma ET (Chroma’s sputter coated) laser blocked (1 mm thick) filter. ET filters achieve the highest transmission levels for interference filters as they have very steep transitions from peak transmission to high optical density to best exploit the spectral properties of modern fluorochromes with short Stokes shifts. This is because steep transitions allow for very little spectral separation between excitation and emission bands. This system has single band (405/488/561/640) ET emission filters, and one dual band (488/561) filter located in a six-position emission filter wheel installed in the CSU-X1 confocal head. Chroma has developed a proprietary manufacturing method that minimizes surface curvature, and all their laser blocked filters have 1 mm thick substrates to ensure surface flatness.

#### Important things to consider for spatial and temporal resolutions

• *Spatial Resolution*: The optical slice size, the number of slices, and the full Z-stack size depend on the size of the objects that is being imaged:
   Optical slice size: The average size of our micro-autophagy event is **0.82 μm ( ±0.23)**.
  ○ This means that the maximum size of a slice size should be ∼0.25 μm to be able to capture the whole event with 3 slices (Methods 2 and 3).
  ○ With Method 1, we used 0.5 μm Z increments, which is not enough for adequate Z resolution. This Z increment was used for capturing most of the vacuole and events occurring in 10 Z steps, which takes 5 s using the two lasers. Using smaller Z increments in this method would result in an increase in the time for acquiring a Z-stack of the same size.
    Z-stack size: The average size of the vacuole is **6 to 10 μm**^**2**^ (−/+ rapamycin, respectively).
    To capture a large part of the vacuole, we took 10 slices (see Table 1).
    ***Note:*** The average size of the vacuole and its increase upon rapamycin treatment we documented (6 to 10 μm^2^) is comparable to the ∼5 μm^2^ reported size, which increases after rapamycin treatment.^7^

**Table 1:** Z-stack setting and duration of a single event

Method	Laser	Slices size (μm)	# Optical slices	Z-stack size (μm)	Interval /Z-stack (s)	Duration of single event (s)
1	Double	0.5	10	5	5	**12.9** ± 3.4
2	Double	0.25	10	2.5	3.7	**8.8** ± 2.0
3	Single	0.25	10	2.5	2.5	**7.3** ± 1.7

• *Temporal Resolution*: Achieving the fastest capture depends on how long it takes to complete a Z-stack. This depends, in order of importance, on (a) two sequential exposure times, (b) changing the filters, (c) Z-motor movement, and (d) changing the lasers. An example is provided.
  ○ Two sequential exposures: exposure time for each channel depends on the signal intensity of the fluorophore:
     Method 1: 200+100 ms = 0.3 s/slice; 10 slices: 3 s.
     Method 2: 200+100 ms = 0.3 s/slice; 10 slices 3 s.
     Method 3: 100 ms/slice; 10 slices 1 s.
    In our current setup, the signals are taken sequentially, and the exposure time is additive.
  ○ Changing the emission filter wheel. During acquisition, this is a rate-limiting step: The physical switch time between two immediately adjacent filters is 90 ms/slice – for a Z-stack (10 slices), 0.9 s. The emission filter switch is controlled by a +5V TTL pulse. If switching between non-adjacent filters the switch time increases by the number of positions away that filter is located.
     Method 1: 2 lasers, 2 filters: The slowest: We used adjacent positions for a faster switch; non-adjacent filters would take longer:
     Method 2: 2 lasers, 1 dual band filter: Faster than Method 1, because there is only changing of the lasers (which is ms), not the filters.
     Method 3: 1 laser, one filter: No changing of lasers and filters gave us the fastest possible Z-stack acquisition, but it was only a single channel.
  ○ Z-motor movement: A negligible factor, and all methods used the same number of slices. 8 ms/step, total for 10 slices: 80 ms.
  ○ Changing the lasers: The most negligible factor. The lasers’ physical response time is 1 ms. The lasers are controlled by a TTL board in the microscope controller box. The power or intensity of the laser line is controlled by an analog voltage that is between 0 and 5 V, this correlates to 0 to 100% for intensity. This analog signal is like the TTL pulse and is generated at the rate of 1 ms. The generation of these pulses is embedded in the 50 ms timing overhead of initiating an acquisition.
    For example: Using Method 2 (2 lasers - single filter): The timing of changing lasers is roughly equal to the exposure time −3 s/Z stack, plus 0.8 s (8 ms per Z Slice) for the Z motor, the computer overhead timing of ∼0.05 s – total ∼3.7 s (Table 1).^1^

## Step-by-step method details

### Cell culture preparation

**Timing: 3–4 days**

Yeast strains are typically stored in glycerol stock at −80°C. The following step explains how to start cell cultures in preparation for live-cell imaging.

1. Streak out the strain(s) of interest on YPD plates. Incubate the plates for 2–3 days at 30°C.

***Note:*** To prevent the plates from drying out, keep them in plastic bags during and after the incubation period at 30°C in an incubator room with a controlled temperature.

2. The day before imaging, inoculate cells from a single colony into 3 mL of YPD medium in a test tube and incubate with rotation at 30°C for 6–16 h.
3. After incubation, measure the OD_600_ from the cell culture.
  a. Dilute it to an OD_600_ of 0.002 into 25–30 ml of YPD in a 50 ml flask.
  b. Incubate the flask at 28°C in an incubator with shaking (200 rpm) 16–18 h up to OD_600_ 0.5–0.8.

**CRITICAL:** Saturated culture is not recommended for this protocol's purpose since it could create additional stress on the cells before the main stress for imaging is induced.

### Nutritional stress induction and lysosome staining

**Timing: 30 min after addition of rapamycin**

Here, we describe steps for the induction of stress using rapamycin and vacuolar staining for live microscopy imaging.

***Note:*** Reason: We determined that clearance of cargo through micro-ER-phagy happens within the first 2 h after addition of rapamycin.^1^ Therefore, we started the acquisition of the videos 30 min after imposing nutritional stress.

4. After the cell culture reached OD_600_ between 0.5 and 0.8, treat cells with rapamycin and transfer 0.5–1 ml of culture into a clean culture tube.
  a. To induce micro-autophagy, treat cells with rapamycin at a final concentration of 200 nM and incubate at 28°C while shaking for 30 min.
  b. For lysosomal staining alternatives:
    i. FM4-64 (lysosomal membrane) was added simultaneously with Rapamycin.
    ii. CMAC (lysosomal lumen) was added 15–30 min before the incubation with Rapamycin end.^5^
5. After staining and treatment, collect cells into 1 ml microtube and spin down at 800 rpm for 2 min.
  a. For FM4-64-stained cells, remove media and wash with 1 ml of YPD + rapamycin once before preparing the microscope slide.^4^
  b. For CMAC-stained cells, there is no need for a wash (according to the dye user manual).

***Note:*** It is important to wash the cells before imaging to remove excess FM4-64 and reduce the background.

6. To prepare the slide for imaging, resuspend cells in YPD + rapamycin by pipetting up and down, and transfer 1.6 μl of stained and treated culture onto the glass slide and cover the drop with a cover glass, size 18 x 18 mm.

***Note:*** No need to sonicate cells at this point (by resuspending cells as described above, no cell clumps are formed).

**CRITICAL:** Make sure there are no air bubbles, which will prevent the cells from settling in the glass slide and will affect the imaging.

7. Load the glass slide with a drop of immersion oil and adjust focus using a 65x oil objective. Should visualize ∼20 or more cells per field of view. The average diameters of a typical cell and vacuole are 5–10 μm and 2–3 μm, respectively.^8^ The average size of an engulfed cargo is 0.82 μm (see below).

***Note:*** As a control, use untreated cells with the same preparation (- rapamycin) for imaging in the next step.

### 4D imaging with confocal fluorescence microscopy

**Timing: ∼55 s to 1 min 30 s for time-lapse acquisition**

The following steps describe the LAS X software setup required for time-lapse imaging of live yeast cells, according to the three methods used in Gyurkovska et al., 2025. No temperature-controlled microscope setup is needed.

8. The setup required for time-lapse imaging depends on the dye used and the time/space resolution desired. The following describes the three methods that can be used (also see Table 1):
  a. Method #1: For dual-channel imaging (double laser acquisition) using Chroma 25 mm single narrow band Emission filters, with FM4-64 dye, the settings for the confocal fluorescent microscope are:
    i. # Z-stacks: 10 every 5 s.
    ii. Z-stack size: 5 μm, 0.5 μm/section.
    iii. Total time of video duration: 90 s.
    iv. Interval: 5 s.
    v. Fluorophores: GFP excitation/emission: 488/505 nm, and FM4-64 excitation/emission: 515/640 nm.
    vi. Laser: Green Channel (cargo) exposure/laser power/wavelength: 200 ms/50%/488 nm, and Near Red Channel (FM464) exposure/laser power/wavelength: 100 ms/20%/561 nm.
  b. Method #2: For dual-channel imaging (double laser acquisition) with Chroma- Dual-band Emitter filter, the settings for the confocal fluorescent microscope are:
    i. # Z-stacks: 10 every 3.7 s.
    ii. Z-stack size: 2.5 μm, 0.25 μm/section.
    iii. Total time of video duration: 57 s.
    iv. Interval: 3.7 s.
    v. Fluorophores: GFP excitation/emission: 488/505 nm, and CMAC excitation/emission: 354/469 nm.
    vi. Laser: Green Channel (cargo) exposure/laser power/wavelength: 200 ms/50%/488 nm, and UV Channel (CMAC) exposure/laser power/wavelength: 100 ms/15%/405 nm.
      ***Note:*** The Chroma dual-band Emitter filter allows faster imaging without the need to change the filter between channels, thus increasing acquisition speed.
  c. Method #3: For single channel imaging (single laser acquisition) using Chroma 25 mm single narrow band Emission filters, with FM4-64 dye, the settings for the spinning disk confocal fluorescent microscope are:
    i. # Z-stacks: 10 every 2.5 s.
    ii. Z-stack size: 2.5 μm, 0.25 μm/section.
    iii. Total time of video duration: 55 s.
    iv. Interval: 2.5 s.
    v. Fluorophore: FM4-64 excitation/emission: 515/640 nm.
    vi. Laser: Near Red Channel (FM464) exposure/laser power/wavelength: 100 ms/20%/561 nm.
      ***Note:*** Acquiring a single snapshot of dual-channel (double laser acquisition) image before and after a single-channel time lapse allows tracking the movement of the cargo. The focus of the image should be in the middle of the Z-stack.
      ***Note:*** For each method, take 5–7 videos with different fields of view per sample.
9. After acquiring the time-lapses, the 3D reconstruction can be performed using the 3D viewer in the LAS X software.

## Expected outcomes

We had two main expectations for using the three methods of this protocol: First, to visualize individual micro-autophagy events in live cells, and second, to get an estimate of how long such events take. For either, we needed high spatial and time-temporal resolutions. Using the methods described above, we could visualize ∼34% of cells going through invagination of the lysosomal membrane.

### Visualization of micro-autophagy events

**Figure 1:**
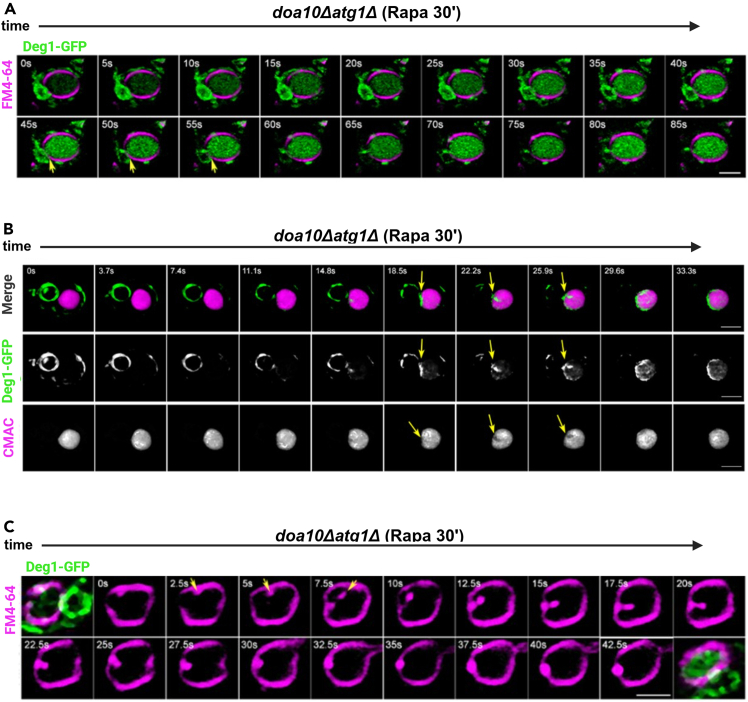
Time-lapse 3D fluorescence microscopy of individual micro-autophagy events: three approaches to improve temporal and spatial resolution Time-lapse microscopy of *doa10Δatg1Δ* cells expressing Deg1-Vma12-GFP (green), 30 min after addition of rapamycin, using two different lysosomal dyes for membrane and lumen. (A) Two fluorophores: 5 s, 0.5 μm/section: Lysosomal membrane stained with FM4-64 (magenta), video acquisition: double laser and double filters. Arrows points to the entry of GFP into the lysosome. Can visualize cargo internalization to the lysosome through the video, but temporal and spatial resolutions are insufficient to determine the duration of single events. (B) Two fluorophores, 3.7 s, 0.25 μm/section: Lysosomal lumen stained with CMAC (magenta); video acquisition: double laser with multi-path filter. Arrows point to the site of GFP entrance to the lysosome and invagination site. The best temporal resolution for using 2 fluorophores is possible with a multi-path filter. (C) One fluorophore, 2.5 s, 0.25 μm/section: - Time-lapse still of lysosomal membrane stained; video acquisition: single laser. First and last frames show snapshots of GFP + FM4-64 at the beginning and end of the video, respectively, to show that the cargo went inside. Arrows points to the invagination site. Improved temporal and spatial resolutions, but only one fluorophore for the video. Scale bar, 2 μm. Figure is an adaptation of Figures S2 and S3 of Gyurkovska et al.^1^ The average time of individual events for each method was 12.9 s ( ±3.4) (A), 8.8 s ( ±2.0) (B), and 7.3 s ( ±1.7) (C). Results in this figure represent three independent experiments.

Figure 1 shows 3D reconstruction of single cells taken in the specified intervals as shown in Gyurkovska et al., 2025.

Method #1 used a double laser and allowed visualization of the GFP-tagged cargo and the lysosomal membrane simultaneously (Figure 1A, arrows point to individual events). During the span of the video, the GFP fluorescence disappears from the cytoplasm and accumulates inside the lysosome, meaning the cargo is going through the invagination of the lysosomal membrane to be degraded inside of the lysosome. While this method allows tracking of the cargo, it does not give the best spatial and time resolutions for capturing a single invagination event.

In Method #2, the use of a double laser with another lysosomal dye allows the visualization and tracking of an invagination event with the cargo at better spatial and time resolutions than in Method #1 by a combination of a fluorescent dye that stains the lumen instead of the lysosomal membrane, and a complementary Chroma- Multi-pass Emitter filter. This method allowed visualization of the cargo entering the lysosomal lumen for degradation (Figure 1B, arrows point to individual events).

With Method #3, the use of a single laser to visualize the movement of the lysosomal membrane allows capturing a single event, starting with the invagination of the membrane and the formation of the lysosomal membrane body inside of the lysosome because of the closure of the membrane (Figure 1C, arrows point to individual events). Capturing the cargo immediately before and after the video ensured that the membrane movement we followed resulted in cargo entering the lysosome.

### Estimate of how long micro-autophagy events take

Using our microscope setup to its limits of spatial and temporal resolutions, we determined that the average size of the events we observed is **0.82 μm.**

With spatial resolution of 0.25 μm/optical slice (Methods 2&3) the event can be seen within 2–3 slices. For example, with 0.5 μm/optical slice (Methods 1), we would miss some events of 0.82 μm size. For 0.82 μm events, the average duration of a single event is **7.3 s** when using 2.5 s per Z-stack acquisition (Method 3). This means that we could observe a single event by acquiring 3 Z-stacks. With Method 2, with 3.7 s per Z-stack acquisition, estimating a duration that takes 7.3 s, would be less accurate (8.8 s).

The remaining question is: if we could use higher spatial and temporal resolutions, would we observe events of higher speed? We think that it is possible that with the spatial resolution we used, we could miss events that are smaller than 0.82 μm, and such events probably would take less time, which we could measure with higher temporal resolution (see troubleshooting below).

Therefore, we conclude that for events that take about 10% of the vacuolar size **(∼8 μm)**, the estimate of **7.3 s** duration is correct.

***Note:*** In LAS X for Z stack acquisition, the default setting is system optimized for the objective being used. The system optimized is Nyquist calculated for the specific objective being used. The system optimized Z stack setting can be overridden by either manually defining the Z slice thickness or manually defining the number of slices. Nyquist was used for all normal imaging. To increase the temporal resolution, a slightly lower number of Z slices for a defined volume was used to increase speed. This was optimized empirically so that when the Z stack was rendered in the 3D viewer, there was no loss of fidelity in the 3D Z render.

## Limitations

Although the methods described above can be used to determine the speed and visualization of micro-autophagy events, each method has its limitations that need to be considered. Because each method has its limitations, it is better to use all three of them before making conclusions.

Method #1 allows the use of both lasers and visualization of both the cargo and the lysosomal membrane; the spatial resolution cannot be increased without affecting the time resolution. Each micro-autophagic event occurs at ∼8 s, meaning that with Method #1, there will be missing information since the lowest time for each frame is 5 s.

Method #2 allows imaging both cargo and lysosome without interfering with spatial and time resolution by using both lasers but changing the lysosomal dye. This method allows image the lysosomal lumen and the cargo getting delivered inside, but it cannot show the invagination of the lysosomal membrane. Because of this, Method #3 was designed.

Method #3 has the highest spatial and time resolution because only one laser was used. This prevents tracking the cargo while it gets invaginated by the lysosomal membrane.

## Troubleshooting

Here, we define four possible problems and provide suggestions for how to address them.

### Problem 1

Bleed through the laser of one channel to the other channel (step 8).

### Potential solution

To prevent this, make sure that each wavelength of your fluorophores/dyes does not overlap. Test each color channel to confirm there is no bleed through.

### Problem 2

Yeast cells move on the slide, preventing accurate imaging (step 6–7).

### Potential solution

Seal the cover slide with nail polish on the edges or use concanavalin A to attach the cells to the slide. Also, load the minimum amount of culture possible on the slide.

### Problem 3

Acquisition with two fluorophores decreases its speed, missing out on information about the duration of the event (step 8).

### Potential solution

Use Chroma -Multi-pass filter that allows the use of two light sources without the need for changing the filters. This will expedite the Z-stack acquisition, preventing the loss of information about the duration of the event.

### Problem 4

For events smaller than ∼0.8 μm and/or faster than ∼7 s (see expected outcomes above), the spatial and temporal resolutions we describe might not be enough. Can the confocal be pushed to higher spatial and temporal resolutions (step 8).

### Potential solutions

The four main ways for increasing acquisition speeds with our current confocal system are: (a) adding a two-camera splitter and a second camera, (b) getting a higher speed piezo Z motor, (c) using stronger fluorophores, and (d) increasing the camera sensitivity. These can also be combined.

• Adding a second camera to capture the two signals simultaneously (and not sequentially as they are captured now) will cut down the exposure time by half. In this setup, the two lasers fire at the same time, the spinning disc acquires one image, a splitting tube (image duplicator) sends the image to two narrow band emitters that determine which signal will be sent to the camera, the images from the two identical cameras can be then overlayed.
• Adding a faster (piezoelectric) Z motor to the system would eliminate the current Z motor delay to move to the next position. This would allow acquiring smaller optical slices, which would improve the spatial resolution.

The two-camera system in conjunction with a faster Z motor setup would bring the system to maximal speed with near real-time view of the events as they unfold.

• The fluorescence intensity depends on the dye, the tag, or the level of the tagged protein, which are all dependent on cellular processes followed. Stronger signals require shorter exposure time.
• Our current camera has a quantum efficiency of 95% (only 5% photon loss), which is a leading-edge technology; however, unknown future technology may offer even shorter exposure times with increased sensitivity. Existing intensifying cameras, which boost the signal through an electronic method, would lower exposure time.

## Resource availability

### Lead contact

Requests for further information and resources should be directed to and will be fulfilled by the lead contact, Nava Segev (nava@uic.edu).

### Technical contact

Questions about the technical specifics of performing the protocol should be directed to and will be fulfilled by the technical contact, Yaneris M. Alvarado-Cartagena (yalvar21@uic.edu).

### Materials availability

All unique/stable reagents generated in this study are available from the lead contact with a completed materials transfer agreement.

### Data and code availability

All data reported in the original paper by Gyurkovska et al.^1^ are available as open access; any additional information required to analyze the data reported here is available from the lead contact or technical contact upon request.

## Acknowledgments

This research was supported by grant GM 141479 from the 10.13039/100000002NIH to N.S. Figures were created using Biorender.com.

## Author contributions

Study conception and design, N.S. and C.H.; data collection, Y.M.A.-C.; data analysis and interpretation, Y.M.A.-C. and N.S. All authors helped with preparation of the manuscript draft. The figures and final manuscript were completed by Y.M.A.-C. and N.S.

## Declaration of interests

The authors declare no competing interests.
